# Trancendental Quackery

**Published:** 1870-11

**Authors:** 


### Trancendental Quackery.

Dr. Acworth, of Brighton, England, is a practitioner of high potencies, whose faculty of credulity has been developed to such an extent by his profound study of Hahnemannian metaphysics that he gravely publishes in the *British Journal of Homœopathy*, an "Account of Count Mattei's Marvellous Medicines," the following summary of which we extract from the *Chemist and Druggist*:—

Count Cæsar Mattei, we are told, is a wealthy nobleman of Bologna. He is an amateur physician, and having thoroughly studied the art of healing, throwing into it all his heart and soul, has now mastered it to such an extent as to leave far behind all the attainments and successes of every other school of medicine which has ever existed. His discovery consists of seven medicines for internal administration, and four for external application. The former are described as follows:—No. 1 cures all manner of coughs, catarrhal and bronchial affections, and incipient phthisis,; 2 is a specific for intermittent fevers, and very useful in case of typhus; 3 sets right the circulating system if disordered, certain diseases of the heart, hæmoptysis,, and many other complaints: 4 is termed *Anti-Canceroso*—this is one of the most completely triumphant of the series,: "Count Mattei counts his cures (of cancer) by scores;" 5 is *Anti-Scrofuloso*; 6 *Anti-Venereo*; 7, *Antic Verminoso.* (The last is a vermifuge, not a vermin killer). This completes the materia medica, as far as internal remedies are concerned. The doses are homœpathic, and the Count has found that the more infinitesimal the dose, the more wonderful is the effect. But the external remedies are by far the most miraculous. We said there were four; in reality they are only one in kind, but are supplied in four degrees of strength extinguished by various colors. Now the internal medicines are secret; the Count, for some philanthropic motive, which we do not quite understand, has sworn not to reveal the mode of preparation, "till their virtues shall be universally acknowledged, and allowed to be superior to those of any now in use." But with the application he is less reticent; there is no secret in this; it is simply liquid elertricity! It is quite immaterial to therapeutics, though it would be interesting from a scientific point of view, to be told how this electricity is got into the fluid state. Dr. Acworth has some of the marvelous article in his possession, and yet all the description of its physical properties which he vouchsafes to an inquiring world is that it is a "c[illegible]lo[illegible]less fluid." The weakest kind sent out by the Count is of the natural color, but it may be had more condensed, and tinted red, green or yellow, to distinguish the various stages of strength. The yellow is a dreadful thing to have about you; we presume it is the essence of forked lightning; and we are cautioned under no circumstances to apply it to the head.

The fact that we are living in the nineteenth century might lead us at first to suppose that such an article written by a man who holds a medical degree, could only be intended as a pleasantly ironical way of describing the vagaries of a recognized lunatic, or the impostures of a self-convicted knave. But no; Dr, Acworth finds confidence in the circumstances that the Count has no mercenary motives

He is wealthy, and has built a hospital in Balogna especially to employ these medicines. When he first commenced to introduce them, he gave them away, and Dr. Acworth seems to think he intended to persist in this suicidal course, if it had not been for the conduct of certain wicked chemists who took advantage of the Count's liberality. "So n[illegible]w, to prevent their being tampered with, the medicines are sold in globules, at such low price that the poor can easily obtain them." Afterwards we learn that the electricity is sold at a hundred francs the litre, which, we expect, helps to defray the expenses of the hospital.

Twenty thousand cases, more or less, of desperate maladies are said to have been cured at the said hospital in the summer of 1867, of which 150 are recorded in a little book in Dr. Acworth's possession, and to which he attaches implicit faith on the strength of his own corroborative experience. In all the multitudinous cases treated, not a single failure is recorded, Count Mattei's Marvelous Medicines being in this, as in some other respects, on a par with the "University Remedies" evolved from the moral consciousness of our own city's modern "Great Unknown."

The only question of much medical interest in such a connection is whether it is Count Mattei or Dr. Acworth who is mad, or both or whether the favorite epithet of our temperate contemporary, the *Tribune*, should be applied to them, or either of them, separately or conjointly.—*Med. Gazette.*

